# Operational derivation of Boltzmann distribution with Maxwell’s demon model

**DOI:** 10.1038/srep17011

**Published:** 2015-11-24

**Authors:** Akio Hosoya, Koji Maruyama, Yutaka Shikano

**Affiliations:** 1 Department of Physics, Tokyo Institute of Technology, Tokyo, 152-8551 Japan; 2 Department of Chemistry and Materials Science, Osaka City University, Osaka, 558-8585 Japan; 3 Research Center of Integrative Molecular Systems (CIMoS), Institute for Molecular Science, National Institute of Natural Sciences, Okazaki, 444-8585 Japan; 4 Institute for Quantum Studies, Chapman University, Orange, CA 92866, USA; 5 Materials and Structures Laboratory, Tokyo Institute of Technology, Yokohama, Kanagawa, 226-8503 Japan

## Abstract

The resolution of the Maxwell’s demon paradox linked thermodynamics with information theory through information erasure principle. By considering a demon endowed with a Turing-machine consisting of a memory tape and a processor, we attempt to explore the link towards the foundations of statistical mechanics and to derive results therein in an *operational* manner. Here, we present a derivation of the Boltzmann distribution in equilibrium as an example, without hypothesizing the principle of maximum entropy. Further, since the model can be applied to non-equilibrium processes, in principle, we demonstrate the dissipation-fluctuation relation to show the possibility in this direction.

Statistical mechanics has been developed in order to describe the behavior of systems that have a large number of microscopic degrees of freedom so that it is consistent with thermodynamics^1^. While it is no doubt the best theory we have today to explain the dynamics of such systems, its foundations are not as solid as they may appear. Particularly, the principle of equal *a priori* probabilities, or the ergodicity of the system, lacks a clear physical rationale, which led to coexistence of various approaches on which the theory is based^2^,^3^,^4^,^5^. The history of each school can be found in e.g., ref. 6, and also in the references of a more recent research paper^7^. This situation is not very comfortable also from the standpoint that physical laws should be constructed based on physical operations, even in a thought experiment, as in the Newtonian mechanics, electromagnetism, and the theory of special relativity.

Thermodynamics, on the other hand, is constructed upon firmly established empirical and operational evidence on macroscopic objects^8^. Further, it is believed to explain a variety of physical phenomena, regardless of the details of the system constituents. Thus we take the universality and robustness of thermodynamics as a guiding principle in our attempt to lay the foundations of statistical mechanics^9^,^10^.

Our motivation is in describing physics in terms of operations, i.e., under the concept of operationalism^11^,^12^,^13^,^14^. In this respect, we need the notion of probability in the consideration to bridge thermodynamics and statistical mechanics and it should be introduced through operations. Fortunately, from the viewpoint of the frequentism^15^, probabilities can be defined as a limit of relative frequencies of events in a large number of trials or operations. Then, the standard information theory^16^,^17^ can fit in the argument based on operations naturally, since the amount of information, such as the Shannon entropy^18^, is defined through probabilities.

Moreover, information processing can also be seen as a physical operation, since once information is encoded in a physical state any computational manipulation is realized as an operation on the state^19^,^20^. This way, we can construct an operational scenario, incorporating the notion of probability via information with thermodynamics^21^.

As a concrete example, here we consider the derivation of the Boltzmann distribution in the canonical ensemble. Perhaps its most notable derivation using the concept of information (or entropy) is the one by Jaynes^22^, who claimed the principle of maximum entropy (PME). Jaynes identified the equilibrium as the state that maximizes the Shannon entropy with respect to the probability of each microscopic configuration under the constraint on the total energy.

While Jaynes’ approach has been very successful, the PME is essentially based on the principle of equal *a priori* probabilities (Bayesian view of probability). This means that no operations are involved in the *a priori* probabilities for the premise of the PME, unlike in those of frequentism.

More recent work that may be relevant is the formulation of the canonical ensemble in the language of quantum mechanics^23^,^24^. They showed that the state *ρ* of a small system is approximately equal to the canonical state *exp*(−*H*/*k*_*B*_*T*), as a result of entanglement between the system and its environment, provided the interaction between the system and the environment is weak. Here, *H*, *k*_*B*_, and *T* are the Hamiltonian of the system, the Boltzmann constant, and the temperature of the environment. Their results are very smart and elegant in their own right, however, they have assumed the *a priori* equiprobability and it is still unclear whether the consideration of quantum entanglement is requisite for the foundations of statistical mechanics.

In this paper, we derive the Boltzmann distribution for the canonical ensemble in an operational manner, i.e., constructing an operation-based scenario, with which we define a function to discuss equilibrium. This approach is useful to clarify the role of information, albeit implicit, in what we already see as a common sense in physics.

A key ingredient in our work that brings the notion of information into physics is information processing, or more specifically, information erasure. The physics of information erasure clarified the link between thermodynamic and information-theoretic entropies^25^,^26^,^27^,^28^,^29^,^30^,^31^, and it played a central role in resolving the paradox of Maxwell’s demon. It states that the erasure of one bit of information (in the demon’s memory) requires a work consumption of at least *k*_*B*_*T* ln 2. Here, *k*_*B*_ is the Boltzmann constant and *T* is the temperature of the heat bath with which the memory system is in contact. Incidentally, despite the extremely small value of *k*_*B*_  ln 2, which is roughly 1 × 10^−24^ J/K, strong experimental evidence for the information erasure principle has recently been reported^32^,^33^,^34^,^35^. If the information content in an *N*-bit string is *NH*(*p*) < *N*, where 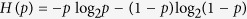 is the Shannon entropy, then the minimum work for erasure becomes *Nk*_*B*_*TH*(*p*) ln 2, as shown in ref. 21. This is because the optimal data compression makes the length of the string from *N* to *NH*(*p*), and after this compression we erase information in the *NH*(*p*) bits in which 0 and 1 appear with equal probability, spending *Nk*_*B*_*TH*(*p*) ln 2 of work.

Here, we make the demon play as a symbolic entity that carries out operations, as we shall present below. Also, because the definition of equilibrium is independent of operations, our scenario has a potential to be applied to nonequilibrium statistical mechanics, as we will describe briefly, taking the fluctuation-dissipation theorem^36^ as an example.

## Result

Let us clarify first what we mean by “Maxwell’s demon”, as sometimes this can be a source of confusion. We basically follow the original idea by Maxwell^37^, although our demon does not intend to violate the second law of thermodynamics^38^,^39^,^40^,^41^,^42^.

In this paper, the demon is an entity that can measure and change the energy levels of particles, and manipulate/process information encoded in memory registers (cells). As it will be clearer below, the particles can have only two distinct energy levels and this is what the demon measures and handles. The demon can of course access the heat bath, thus extract and discard energy from/to it via appropriate tools, complying with the laws of thermodynamics.

The memory is embedded on a long tape, as in the Turing machine that is an abstract, but common, model for information processing. The tape can also be used as a working space for computation, if necessary.

We note that the demon should be able to work autonomously, once the protocol and algorithm for its task are given. The phrase “autonomous system” may refer to a system consisting of mechanical parts that is designed to work on its own (without energy supply or active control from outside), e.g., a Szilard-engine-type machine presented in ref. 43. Nevertheless, for our purpose, it is sufficient to consider a system that proceeds deterministically reacting to the input from outside, complying with physical laws. Naturally, in order to work independently, it should not be fed any extra information or energy as a whole.

So, the name “demon” has merely a symbolic meaning here; it can be replaced with a machine that is capable of storing and processing information, and manipulating the particle states. Although it could be done with some inspiration from an example in ref. 43, devising such a structure in detail is out of our scope and would be left for future work.

### Thermo-Turing model

The primary components of our model are a set P of *N* particles with two energy levels *E*_0_ and *E*_1_(>*E*_0_) and a long tape M, which represents the demon’s memory and contains a sequence of *N* memory cells. We let Φ_0_, Φ_1_ and *Ε* denote the two (ground and excited) states of the particles and the energy gap *E*_1_ − *E*_0_, and assume that each particle is numbered to make a correspondence with a memory cell. The memory tape M can be thought of as a part of the demon and it is very similar to the one we typically consider in the context of Turing machine. Each memory cell can store a binary information, either 0 or 1, and it can be modelled as the Szilard engine^44^, which is a one-molecule gas with a partition at the center of cylinder. We call the mechanism comprising of M and the demon a “thermo-Turing model” in the following discussion.

In the context of (thermodynamic and algorithmic) entropy from the operational point of view, Zurek considered a model of demon with a Turing machine in ref. 28. Here, we incorporate the notions of information processing a la Turing and of thermodynamic consideration of Maxwell’s demon to step in the field of statistical mechanics.

**Figure 1:**
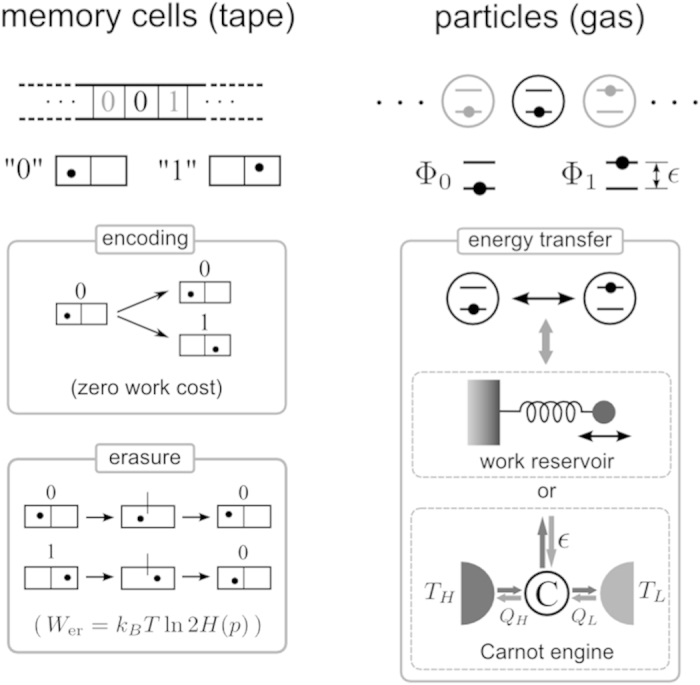
Elements of the thermo-Turing model. Two basic operations on the memory cells, encoding and erasure, are depicted on the left. Each memory cell is modeled with a single molecule gas contained in a cylinder. Encoding is carried out by slowly displacing the region of volume *V*/2 in which the molecule is kept, thus no work is consumed, or by simply rotating the cylinder by 180 degrees when the value is one. Information erasure requires a work consumption of *k*_*B*_*T*  ln 2*H*(*p*), where *H*(*p*) is the amount of information stored in the tape. The two-level particle system is sketched on the right. Energy transfer to the particles can be done with a work reservoir and an unspecified mechanism, or with a fictitious Carnot engine. All these operations on the memory cells and the particles are controlled by the demon (not shown).

In our thought experiment, the interaction between P and the heat bath is mediated by the demon (or the thermo-Turing machine). The rough idea is as follows. The interaction with heat bath causes noise on P and an energy change in it. The degree of noise depends on the bath temperature T, but we represent it only by probability *p* of a state flip. Equilibrium is defined as the state in which the energy change in P is balanced with the energy consumption for subsequent manipulations of the memory tape M at T. Thus, the temperature T comes in to the discussion explicitly only through demon’s actions on P. Note that it is legitimate to assume that M is designed to make the stored information insensitive to thermal fluctuation. This picture (of having a direct effect of T on M) may appear strange from the viewpoint of the conventional deductive approach. However, this scenario allows us to use the demon as a subject of physical ‘operations’ to bring thermodynamic notions into the discussion. The elements of the thermo-Turing model and basic operations therein are depicted in Fig. 1.

Naturally, we consider the memory tape M to let it reflect the state of particles in P. Suppose a situation in which the fraction *p* of a set of *N* particles are in the excited state, i.e., *pN* particles are in Φ_1_, while (1 − *p*)*N* in Φ_0_. Let *F* be the amount of work that the entire system (P + M) can potentially exert towards the outside, when we let it be in the state where all particles are in Φ_0_ and all memory cells store ‘0’. It simply means that we take the state with all in Φ_0_ and ‘0’ as the origin for the quantity *F*.

The energy stored in P contributes to *F* positively, and its amount is 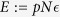. On the other hand, in order to erase all information on the tape, we need to consume some energy *W*_er_. As a result, we have




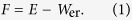




Since we are naturally interested in the optimal (largest) value of *F* for a given *p* in order to characterize the state uniquely, *W*_er_ needs to be minimized. Thus, we have *W*_er_ = *NH*(*p*)*k*_*B*_*T* ln 2^21^, which leads to




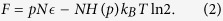




Equation (2) resembles the Helmholtz free energy, i.e., *F* = *U* − *TS*, however, the conceptual difference behind them should be emphasized. The point is in presenting the operational scenario for statistical mechanics by identifying the thermodynamic entropy with the information entropy.

With the definition of *F*, which is computable for any physical state, we shall now define the equilibrium in terms of *F*. We call that the state is in equilibrium when its *F* is stationary, i.e.,




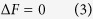




against small noises on the particles. We consider the NOT (flipping) operation on a particle as an elementary process of the noise, thus Eq. (3) is a condition against a small number of random NOT operations on P. This definition of equilibrium is associated with the stationarity of the principal system and the memory tape, rather than the largest likeliness of the state as in Jaynes’ argument^22^. Our definition fits the operational point of view better, because the quantity *F* can be computed by considering physical operations. The operational process (by the demon) will be presented below, soon after deriving the expression of the Boltzmann distribution. Also, a comparison with Jaynes’ work is given in Supplementary Material.

Let us compute Δ*F* for a probability change from *p* to *p*′.




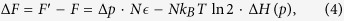




where Δ*p* = *p*′ − *p* and Δ*H*(*p*) = *H*(*p*′) − *H*(*p*). Since the number of errors is small (Δ*p* ≪ 1), Δ*H*(*p*) = *dH*(*p*)/*dp*Δ*p* ≡ *H*′(*p*)Δ*p*. The equilibrium condition, Δ*F* = 0, gives




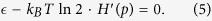




Suppose that the probability change, *p* → *p*′, is induced by thermal noise that flips the state of a randomly chosen particle in P between Φ_0_ and Φ_1_. Noting that ln 2 ⋅ *H*′(*p*) = ln[(1 − *p*)/*p*], we see that Eq. (5) reduces to




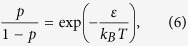




which is nothing but the Boltzmann distribution. The generalization of the model to *d*-level physical systems is presented later in this section.

A similar analysis on the effect of the Toffoli gate is also insightful, however, it is summarized in the Supplementary Material so that we focus on the derivation of the Boltzmann distribution here.

**Figure 2:**
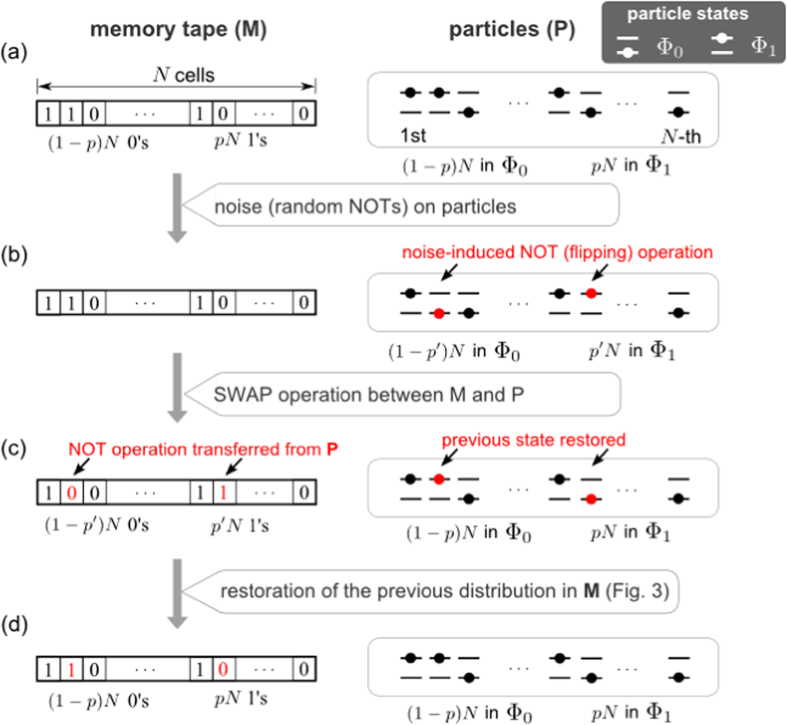
The virtual process for which we consider the change of *F*. (**a**) Each memory cell has a perfect correlation with the corresponding particle’s state. (**b**) The interaction between the particles and heat bath causes a NOT (=flipping) operation to a small number of particles randomly. (**c**) The demon swaps the information stored in the memory and the particle state, e.g., 0-Φ_1_ becomes 1-Φ_0_, and vice versa. (**d**) Both M and P return to the original state that is the same as (**a**).

Next, we describe the operational scenario that naturally leads to the equilibrium condition, Δ*F* = 0 with Eq. (4). Figure 2 depicts the process.

**Figure 3:**
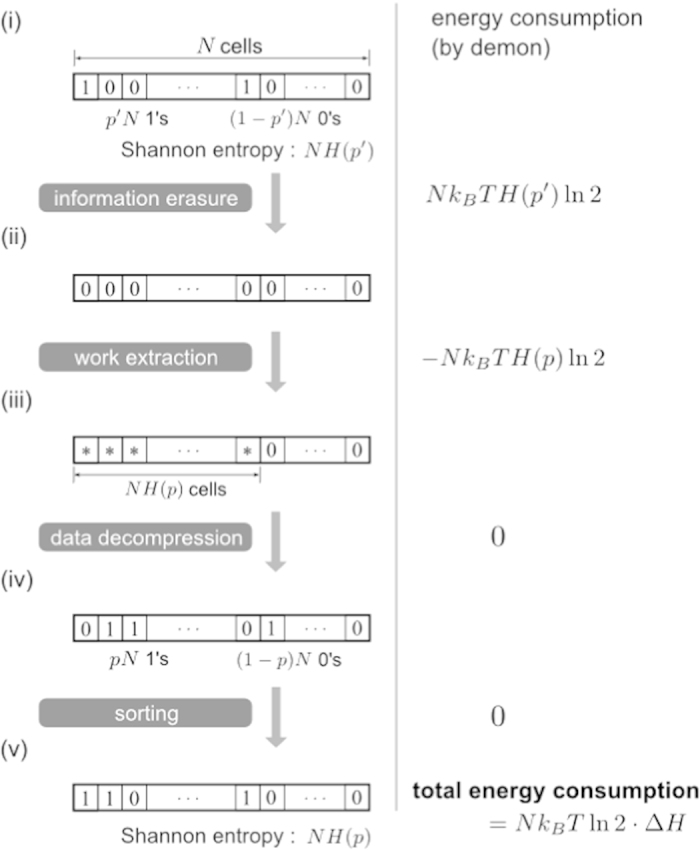
State transformation to change the probability distribution from *p*′ to *p*. All memory cells are reset to ‘0’, consuming *k*_*B*_*T*  ln 2*H*(*p*′) of work (from (i) to (ii)). In (iii), the one-molecule gases in *NH*(*p*) cells are expanded isothermally, giving the demon *k*_*B*_*T*  ln 2*H*(*p*) of work. The ‘*’ sign in (iii) represents a randomized memory state with no physical distinction between 0 and 1; the molecule can move around in the whole configuration space of the cylinder. Since the state in (iii) is the same as the resulting state of data compression for an *N*-bit string containing *NH*(*p*) bits of information, data decompression leads to the string in which *pN* bits are in ‘1’ and the rest are in ‘0’ as in (iv). By permutating the bit string, which can be done for free of energy, the memory tape with *NH*(*p*) of information and the perfect correlation with the particles’ state can be realized.

- One-to-one correspondence between the data stored in the memory tape M and the state of each particle in P is established. That is, if a particle is in the ground state Φ_0_ the corresponding memory cell stores ‘0’, and if the particle is in Φ_1_ the memory has ‘1’. This correspondence can be made by measuring the particle state and copying the result to the memory, which can be done without energy consumption^26^,^27^.
- During some time interval Δ*t*, a NOT (flipping) operation is applied to a few randomly chosen particles. This may be induced by noise or thermal fluctuation, i.e., the interaction between particles and the heat bath. Since the interaction with heat bath is not under the demon’s control, he spends zero energy here. Δ*t* can be taken so that the number of flips is much smaller than *N*.
- The demon swaps the states of particles and memory registers in the tape. The effect of the NOT operations in (b) is now transferred to the tape, while the state of particles is restored to be the one in (a). The energy of 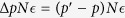 is acquired by the demon to change the particle state, while the SWAP operation for information can be performed without energy consumption as it keeps the entire entropy unchanged. M now has the Shannon entropy *H*(*p*′).
- The demon transforms the Shannon entropy of M from *H*(*p*′) to *H*(*p*). This can be done by the process depicted in Fig. 3, which is explained in detail below. The energy required for this entropy transformation is *Nk*_*B*_*T* ln 2 ⋅ Δ*H* := *Nk*_*B*_*T* ln 2(*H*(*p*′) − *H*(*p*)).

The resulting state in (d) of the above process is the same as (a), and all steps can be made completely autonomous. That is, no traces of the actions by demon are left not only inside M and P, but also in their surrounding environment, while the only possibility of the trace is the amount of energy the environment received. Therefore, for the the joint thermo-Turing system M + P to be in equilibrium, i.e., no macroscopic change detectable from outside, the energy transfer between the joint system and the environment should be zero. Indeed, this condition can be written as




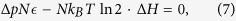




which is Δ*F* = 0.

Figure 3 shows the process to change the state of memory tape so that its entropy is transformed from *H*(*p*′) to *H*(*p*). Incidentally, this process can be seen as a special (classical) case of the one in Fig. 2 of ref. 45, which presented a thermodynamical transformation of quantum state from *σ* to *ρ*. Here, let us proceed with Fig. 3 solely.

From Fig. 3(i) to (ii), the demon erases all the information stored in the tape, consuming at least *Nk*_*B*_*T* ln 2 ⋅ *H*(*p*′) of work.

In Fig. 3(iii), the demon extracts *Nk*_*B*_*T* ln 2 ⋅ *H*(*p*) of work from the heat bath by letting the gas in *NH*(*p*) cells expand isothermally, which is possible since each memory cell can be modelled by a one-molecule gas. Note that the demon can always have the values of *p* and *p*′ since measurement can be done for free. Inserting a partition at center of each cell, now there are *NH*(*p*)/2 cells that represent ‘0’ and another *NH*(*p*)/2 cells ‘1’ (with negligible fluctuation when *N* is large enough).

Then the (Shannon) data decompression^18^ is performed on all the *N* cells to have *pN* cells in ‘1’ and (1 − *p*)*N* cells in ‘0’ as in Fig. 3(iv). Now that the number of cells in ‘1’ is the same as that of particles in excited state Φ_1_ in Fig. 2(d), the demon can sort the order of memory cells to make one-to-one correspondence with the particle states. The sorting process can be done isentropically, thus autonomously, since it is achieved simply by applying an appropriate permutation. Alternatively, a controlled-NOT operation may be applied between M and P, with a memory cell as a control bit and the corresponding particle as a target bit. Because the number of particles in Φ_1_ is the same at Steps (iv) and (v), no extra energy is necessary as a whole.

In summary, we have devised a physical scenario with which we can derive the Boltzmann equilibrium distribution in the statistical mechanics in an operational manner. The operations are performed on the particles and a virtual Turing-machine-type memory cells. We have symbolically used Maxwell’s demon-type intelligent being as the principal operator, but all actions are autonomous and leave no traces observable from outside, thus the demonic actions can be programmed in the Turing machine per se.

The erasure principle, stemming from the paradox of Maxwell’s demon, bridges thermodynamics and statistical mechanics via the notion of probability in information theory. It should be emphasized that we did not base our argument on the equiprobability principle. That is, we did not rely on the standard micro-canonical statistical mechanics, in which the entropy *S* is given by the Boltzmann formula *S* = *k*_*B*_*log*Ω(*E*) with Ω(*E*) being the number of states under a given energy *E*.

Also, the above model can be used to justify the equivalence between thermodynamic and information theoretic entropies, which was discussed in our previous work^21^ in a different context. A brief argument is given in this line in Supplementary Material.

### Generalization to *d*-level system

The above argument to derive the Boltzmann distribution can be generalized to the systems of arbitrary levels. That is, the cells of the tape can store *d* values from 0 to *d* − 1, and there are *d* possible states for particles, Φ_0_, Φ_1_, ..., Φ_*d*−1_, whose energy levels are *E*_0_, *E*_1_, ..., *E*_*d*−1_, respectively. Let *p*_*i*_ be the ratio of the number of particles in the state Φ_*i*_. Suppose that the *k*-th cell of the tape stores the value *i* when the *k*-th particle is excited to Φ_*i*_. This state preparation can be completed by simply copying the measurement result about the particle state.

Instead of the noise-induced random NOT studied above, let us consider random SWAP operations that change the state of a particle. Let SWAP_ij_ denote a SWAP between two states Φ_i_ and Φ_j_, namely, SWAP_ij_ maps the state Φ_i_ of a randomly chosen particle to Φ_j_ and vice versa. Note that the NOT operation between two levels is effectively the same as the SWAP between them, so the process for the thermo-Turing system is basically the same as the one described above (and Fig. 2) with the replacement of NOT with SWAP.

Suppose that a SWAP_*ij*_ has occurred to one of the particles. The SWAP_*ij*_ changes its state if it is in either Φ_*i*_ or Φ_*j*_, otherwise nothing happens. The probability of such a ‘successful’ SWAP_*ij*_ is *p*_*i*_ + *p*_*j*_. After the demon swaps the information between *M* and *P*, the memory tape *M* contains information after the SWAP_*ij*_, and the particles *P* returns to the state before the SWAP_*ij*_ (as in Step (c) above). Thus energy change in *P* due to this operation is (*E*_*j*_ − *E*_*i*_)(*p*_*i*_ − *p*_*j*_)/(*p*_*i*_ + *p*_*j*_) on average.

The change in the erasure entropy times temperature after the single SWAP_*ij*_ and swap between *M* and *P* is




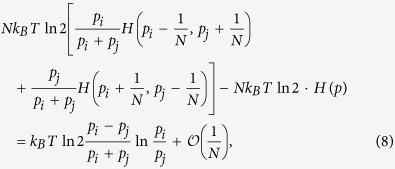




where




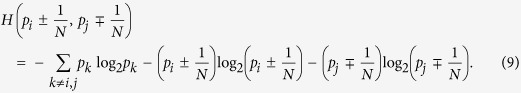




Making the change in *F* equal to zero as in the case of bits and two-level particles, we arrive at the desired relation:




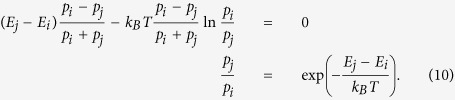




This relation holds for any pairs of *i* and *j*, hence *p*_*i*_ ∝ exp(−*E*_*i*_/*k*_*B*_*T*) for all *i*.

## Discussion

In principle, our thermo-Turing model can be applied to generic non-equilibrium processes, as far as we can assume that the operations by demon can be carried out sufficiently fast, compared with the dynamics. Here, we present a modest step to this direction, choosing a particular model which exhibits a characteristic feature of fluctuation-dissipation theorem.

Suppose that a spatially fluctuating external field that works as a perturbation to energy levels is applied to let the system P deviate from macroscopic equilibrium. This field causes a small change to the energy gap of the particle at the *n*-th site to make it 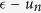, and we assume 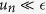 and 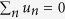 for simplicity. Such a change may be seen as a result of the Stark or the Zeeman effect, but we do not need to specify the origin of the shift for our discussion.

In order to discuss statistical quantities for each particle, the site *n* = 1, 2, …, *N* should be regarded as a block, which consists of sufficiently many members. For the *n*-th block, due to the energy shift *u*_*n*_, the local equilibrium distribution becomes




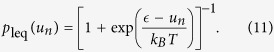




Under this distribution, the operations by demon within the *n*-th block balance with the external field. The index ‘leq’ for *p* in Eq. (11) stands for local equilibrium.

What we are interested in is the amount of dissipation, given the fluctuation of {*u*_*n*_}, 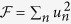. Imagine that the demon now looks at all the blocks as a whole, and attempts to make all particles return to the same equilibrium state, i.e., *u*_*n*_ = 0 all blocks. This is done by changing the energy state of each block and erasing information about the spatial variation of the energy shifts. The work that needs to be done by demon in order to change the distribution *p*_leq_ to that of equilibrium 
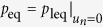
 is




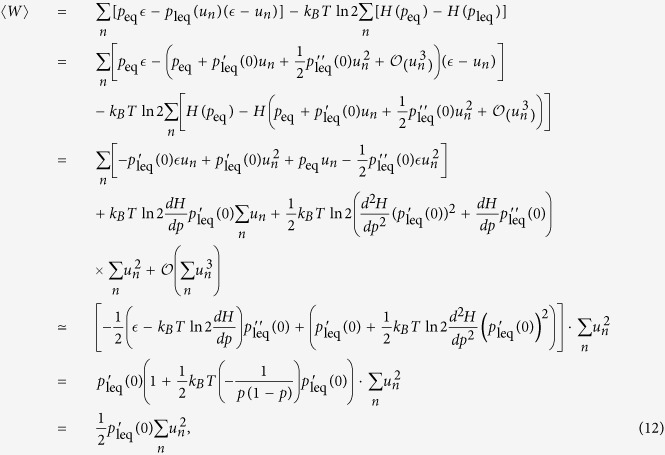




where 
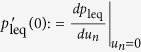
, 
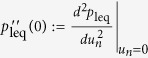
, and 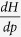 and 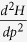 are evaluated at *p* = *p*_eq_. Also, we have used 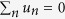 in the fourth equality, and the condition Eq. (5) for equilibrium in the fifth equality. From Eq. (11), we have




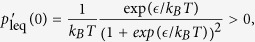




therefore,




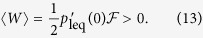




Equation (13) means that the response of the system to the external field results in the positive work by demon, 〈*W*〉 > 0, which is dissipated into the heat bath. Further, it is proportional to the fluctuation of the external field. It is a simple expression of the dissipation-fluctuation theorem in the linear approximation of the fluctuating potential *u*_*n*_. Also, Eq. (13) is interesting in the sense that our model explicitly takes into account of the cost of ‘forgetting the past’, which is simply neglected in the standard consideration of Markovian processes.

The readers who are familiar with the standard dissipation-fluctuation theorem^36^ would feel more comfortable with the fluctuation in time rather than the spatial one of the external potential. In that case, one can reorder the site numbers according to the order of occurrence of *u*_*n*_. Then, the *n* can be interpreted as time, and the average 〈⋅〉 can be understood as that over a long time.

## Additional Information

**How to cite this article**: Hosoya, A. *et al.* Operational derivation of Boltzmann distribution with Maxwell's demon model. *Sci. Rep.* **5**, 17011; doi: 10.1038/srep17011 (2015).

## Supplementary Material

Supplementary Information
