# Supplementary material for: Operational derivation of Boltzmann distribution with Maxwell’s demon model
Source: Sci Rep. 2015 Nov 24;5:17011. doi: 10.1038/srep17011 (PMC4657058; doi:10.1038/srep17011)
Supplement: Supplementary Information [file srep17011-s1.pdf]

Supplementary Material: Operational derivation of Boltzmann Distribution with Maxwell's demon model

Akio Hosoya[1], Koji Maruyama[2], Yutaka Shikano[3,4]

[1]*Department of Physics, Tokyo Institute of Technology, Tokyo, 152-8551 Japan*

[2]*Department of Chemistry and Materials Science, Osaka City University, Osaka, 558-8585 Japan*

[3]*Research Center of Integrative Molecular Systems (CIMoS), Institute for Molecular Science, Okazaki, 444-8585 Japan and*

[4]*Institute for Quantum Studies, Chapman University, Orange, CA 92866, USA*

I. COMPARISON WITH JAYNES' WORK

In the work on the relation between information theory and statistical mechanics, Jaynes derived the corresponding equilibrium state by postulating the principle of maximum entropy[1], which is described as follows. The plausible probability distribution is determined by the requirement that the estimator function $\mathrm{Est}(p_1, p_2, \ldots, p_n)$ is maximized under a given average energy $\langle E \rangle = \sum_i p_i E_i$ with $\sum_i p_i = 1$. Following Shannon[2], he required the mathematical properties for the estimator $\mathrm{Est}(p_1, p_2, \ldots, p_n)$: (i) $\mathrm{Est}(p_1, p_2, \ldots, p_n)$ is a continuous function of p_i, (ii) $f(n) := \mathrm{Est}(1/n, 1/n, \ldots, 1/n)$ is a monotonic increasing function of n, and (iii) $\mathrm{Est}(p_1, p_2, \ldots, p_n)$ satisfies the composition law. From these mathematical requirements (i)-(iii), the estimator is uniquely determined to be equal to the Shannon entropy, i.e., $\mathrm{Est}(p_1, p_2, \ldots, p_n) = -\sum_i p_i \log p_i$. Maximizing the Shannon entropy under the constraints that $\langle E \rangle$ is fixed and $\sum_i p_i = 1$, the plausible probability distribution is obtained to be the one in Eq. (19) of the main text. Note that the temperature does not come in from the physical assumption but is defined so that the free energy thereby obtained coincides with the Helmholtz free energy.

In the present work, we have devised a model consisting of a set of particles (physical system) and a demon with components for information processing, namely a memory tape and a processor. The memory tape is in the contact with a heat bath, and the demon operates them to simulate the effect of interaction between the particle system and a heat bath, so that the particles' energy state will have the right probability distribution. We have defined the equilibrium state operationally and subsequently derived the Boltzmann distribution. Thus, the temperature dependence of the distribution was naturally obtained, whereas it was not so in Jaynes' mathematical work.

II. EFFECT OF TOFFOLI GATES

The effect of the Toffoli gate as an error operation is also worth studying, since errors can be any arbitrary logical operations and it is well known in computer science that arbitrary logical operations can be simulated by NOT and Toffoli gates as far as the operation is logically reversible[3]. The Toffoli gate works on three bits, two of which are control bits and the other one is the target bit. The state of the target particle is flipped when the two control bits are in the excited state ('1' in the tape M), thus the flip of the target is activated with probability pp^*, where $p^* = (pN-1)/(N-1)$ is the probability of finding the second control particle in the excited state Φ_1 when the first one is also in Φ_1. The flip operation on the target is applied to the particle in Φ_1 with probability $p^{**} = (pN-2)/(N-2)$ and the resulting ratio of those in Φ_1 will be $p - 1/N$. Similarly, if the target was in Φ_0 the ratio p will be changed to $p + 1/N$. Therefore, the average change of the Shannon entropy is

$$\begin{aligned}\Delta H &= pp^* \left[p^{**} H\left(p - \frac{1}{N}\right) + (1-p^{**})H\left(p + \frac{1}{N}\right)\right] + (1-pp^*)H(p) - H(p) \\ &\simeq \frac{1}{N}pp^*(1-2p^{**})H'(p) + \frac{pp^*}{2N^2}H''(p), \qquad (1)\end{aligned}$$

where the second term $(1-pp^*)H(p)$ represents the case of no activation of the flip on target.

With the above ΔH, the change in F due to a random Toffoli operation can be computed as

$$\begin{aligned}\Delta F(\text{Toffoli}) &= \Delta p N\epsilon - k_B T \ln 2 \cdot \Delta H \\ &= pp^*(1-2p^{**})\epsilon - pp^*(1-2p^{**})k_B T \ln 2 \cdot H'(p) - \frac{pp^*}{2N}k_B T \ln 2 \cdot H''(p) \\ &= -\frac{pp^*}{2N}k_B T \ln 2 \cdot H''(p) + \mathcal{O}\left(\frac{1}{N^2}\right) \\ &\to -\frac{p^2}{2N}k_B T \ln 2 \cdot H''(p) \quad (\text{as } N \gg 1). \qquad (2)\end{aligned}$$

We have used the equilibrium condition, Eq. (5) in the main text, in the third equality. The effect of the random Toffoli gate is essentially the same as that of the random NOT (corresponding to ΔH with $pp^* = 1$ in Eq. (1)) except for the overall probability factor p^2 for having two 1's in the control bits.

Thus, the sum of the second order contribution from the simple random NOT (=flip) and Toffoli operations is

$$\Delta F = -k_B T \frac{\ln 2}{2N}\left[\#(\text{NOT}) + \#(\text{Toffoli})p^2\right] H''(p) \ (> 0). \qquad (3)$$

That is, the change ΔF by the random NOT and Toffoli gates strengthens the local stability of the cost function F near the equilibrium, which is also expected from the convexity of the entropy

function. This further supports our definition of equilibrium in the thermo-Turing model. It is also worth noting that the quantity in the square bracket is the effective computational complexity[4].

III. NOTE ON THERMODYNAMIC AND INFORMATION-THEORETIC ENTROPIES

In our previous paper[5], using the scenario of Maxwell's demon, we showed that the thermodynamic entropy coincides with the information theoretic entropy in the optimal case of the memory reset. In this section, we attempt to look at the same problem from a different perspective going back to the the basic thermodynamics.

Let us recall how the thermodynamic entropy was introduced in the context of Carnot's theorem. Let the work exerted towards the outside be $W(i \to f)$ for an isothermal state change $i \to f$, which is in general different from the change of the internal energy $U_i - U_f$. An invisible energy flow that contributes to the energy balance is called heat Q exchanged between the system and the heat bath. Thus, the energy conservation is written as

$$W(i \to f) = U_i - U_f + Q. \tag{4}$$

Carnot's theorem claims that the maximal heat flow from the heat bath in isothermal process is proportional to the temperature T of the heat bath,

$$Q_{max} = TS_{th}, \tag{5}$$

where the maximization is made over all possible intermediate processes between the initial and the final states[6]. The coefficient S_{th} is defined as the increase of the thermodynamic entropy.

Now go back to our thermo-Turing model. Clearly, our F in Eq. (2) of the main text is the negative of the work that can be exerted outwards,

$$F = -W(i \to f). \tag{6}$$

Letting the minimum of F in Eq. (6) be equal to Eq. (3) in the main text, we have

$$F_{min} = -W_{max} = -U_i + U_f - Q_{max} = p\epsilon N - NH(p)k_B T \ln 2. \tag{7}$$

Since $-U_i + U_f = p\epsilon N$ in our model, we arrive at the equivalence between the thermodynamic and the information-theoretic entropies;

$$S_{th} = NH(p)k_B \ln 2. \tag{8}$$

It is interesting to see that both the thermodynamic and the information theoretic entropies are defined as a limit, while the former is physically realized in the quasi-static limit and the latter is achieved by the optimal limit of the data compression. The quasi-static processes means that the operation has to be slow enough compared with microscopic processes. This can be viewed as an aspect of the Markovian process, in which the information on the earlier configuration is lost due to, e.g., the multiple-scattering of particles by the cylinder wall. The corresponding process in our model is the erasure of information, which occurs when the partition in the memory cell is removed. To lose the information stored in the memory, we need to wait for a while until it becomes impossible to infer the history of molecule's trajectory.

As a byproduct, we can see that the extensivity of the thermodynamic entropy follows from that of the Shannon entropy. Note also that Eq. (8) can be viewed as the Boltzmann formula $S_{th} = k_B \ln W$, where $W = 2^{NH(p)}$ is the number of possible states under a given total energy.

---

[1] Jaynes, E. T. Information Theory and Statistical Mechanics, *Phys. Rev.* **106**, 620 (1957)

[2] Shannon, C. E. A Mathematical Theory of Communication, *Bell System Technical Journal* **27**, 379; 623 (1948)

[3] Nielsen, M. A., and Chuang, I. L. *Quantum Computation and Quantum Information*, Cambridge University Press, Cambridge (2000).

[4] Zurek, W. H. Algorithmic randomness and physical entropy, *Phys. Rev. A* **40**, 4731 (1989).

[5] Hosoya, A., Maruyama, K., and Shikano, Y. Maxwell's demon and data compression, *Phys. Rev. E* **84**, 061117 (2011)

[6] Tasaki, H. *Thermodynamics* (in Japanese), Baifukan, Tokyo (2000)
